# Editorial: Cardiovascular Fibrosis and Related Diseases: Basic and Clinical Research Advances

**DOI:** 10.3389/fcvm.2022.879780

**Published:** 2022-03-24

**Authors:** Yihua Bei, Hong S. Lu, Jiuchang Zhong

**Affiliations:** ^1^Cardiac Regeneration and Ageing Lab, Institute of Geriatrics, Affiliated Nantong Hospital of Shanghai University, School of Medicine, Shanghai University, Nantong, China; ^2^Shanghai Engineering Research Center of Organ Repair, School of Life Science, Shanghai University, Shanghai, China; ^3^Department of Physiology and Saha Cardiovascular Research Center, College of Medicine, University of Kentucky, Lexington, KY, United States; ^4^Heart Center and Beijing Key Laboratory of Hypertension, Beijing Institute of Respiratory Medicine and Beijing Chaoyang Hospital, Capital Medical University, Beijing, China

**Keywords:** cardiovascular fibrosis, myocardial remodeling, heart failure, pathogenesis, hypertension, therapy, cardiovascular diseases

Cardiovascular fibrosis is a critical pathological process of cardiovascular diseases. Generally, cardiovascular fibrosis arises from enhanced resident immune and inflammatory response, uncontrolled cellular proliferation, and activation of extracellular matrix-producing myofibroblasts due to an aberrant wound-healing response to myocardial injury, ultimately leading to cardiovascular stiffness, pathological remodeling and cardiovascular disorders (1). In this Research Topic “Cardiovascular Fibrosis and Related Diseases: Basic and Clinical Research Advances”, we assembled a collection of 7 original experimental research articles, 3 omics data profiling and integrated analysis articles, and 3 reviews, which address a spectrum of basic and clinical aspects of cardiovascular fibrosis and related diseases.

As the major bioactive peptide of the renin-angiotensin system, Angiotensin II (Ang II) contributes to cardiovascular fibrosis and abdominal aortic aneurysm (AAA). Infusion of Ang II through mini osmotic pumps is a common mouse model to study AAA (2). Consistent with recent reports, Wei et al. found that infusion of Ang II induced neutrophil extracellular trap (NET) formation in the aortic wall of apolipoprotein E (ApoE) deficient mice. Peptidyl arginine deiminase 4 (PAD4) catalyzes citrullination of histones, thereby playing an important role in NET formation in AAA. PAD4 inhibitor YW3-56 improved survival and attenuated maximal diameters of the abdominal aortic region in Ang II-infused ApoE deficient mice. Chen et al. bred PGC-1α floxed mice with Cre transgene driven by SM22, and reported that deletion of PGC-1α aggravated Ang II-induced cardiac fibrosis and injury.

Pathological hypertrophic stress leads to progressive decompensated cardiomyocyte hypertrophy, fibrosis, and heart failure. Zhuang L, Mao Y, et al. provided a novel perspective into the association of fatty acid binding protein 3 (FABP3) with cardiac hypertrophy. They demonstrated a beneficial value of FABP3 in reducing cardiac hypertrophy, fibrosis and heart failure by inhibiting PPARα degradation. Targeting FABP3 represents an attractive approach to prevent cardiac fibrosis and heart failure by alleviating deranged metabolism under hypertrophic stress.

Aging-associated atrial fibrillation (AF) can develop left atrial (LA) dysfunction and heart failure with preserved ejection fraction (HFpEF). Lin et al. demonstrated reduced left atrial (LA) performance and progressively augmented cardiac fibrosis in the aged population, whose alteration was more obvious in AF patients. Intriguingly, a negative correlation has been found between cardiac fibrosis degree and LA performance. Meanwhile, reduced LA performance, enhanced fibrosis, and increased inducibility of AF have been observed in aged mice. Notably, Tan et al. employed the descending aortic constriction (DAC) technique to establish Tibetan minipigs HFpEF model, characterized with cardiac inflammation, hypertrophy and fibrosis and diastolic dysfunction, indicating that DAC-induced porcine HFpEF model is a useful tool to investigate the mechanisms of HFpEF and related cardiac fibrosis.

Cardiac fibrosis is also a common pathology of various inherited cardiovascular and non-cardiovascular diseases. Hypertrophic cardiomyopathy (HCM) leads to microvascular dysfunction, cardiac fibrosis, and ultimately heart failure or sudden death. Cheng et al. addressed novel therapies for HCM based on regulating calcium homeostasis and sensibility in the myocardium. Autosomal dominant polycystic kidney disease (PKD) is a common hereditary renal disorder associated with arrhythmogenic remodeling. In addition to the secondary cardiovascular complications in PKD, Amirrad et al. revealed that PKD2 deficient mouse developed pathological hypertrophy, interstitial and conduction system fibrosis, and cardiac dysfunction with a predisposition to arrhythmia, accompanied with elevated levels of fibrosis-associated TGF-β1 and TGF-β1 receptor.

Due to the complexity of etiology and pathogenesis of cardiac fibrosis, it is highly needed to identify and develop novel therapeutic targets. In the cardiovascular system, microRNAs (miRNAs) regulate signaling pathways in cardiac fibrosis and remodeling (3). Hua et al. focused on the current understanding of miRNA-34a in its biological effects and implications for cardiac pathologies. MiRNA-34a plays a crucial role in diverse cardiac biological pathways that induce cardiovascular fibrosis and dysfunction. Inhibiting miRNA-34a has emerged as a potential therapeutic strategy for cardiovascular fibrosis and related diseases. Iloprost is a synthetic prostacyclin receptor agonist by reducing right ventricular collagen synthesis during pulmonary hypertension. Faggioli et al. discussed the multiple beneficial effects of iloprost on leukocyte functions and inflammatory response in COVID-19 patients with either acute digital peripheral ischemia or ventilation.

High-throughput omics technologies and multi-omics or integrated analysis are powerful tools to screen and find potential regulators in diseases. Zhuang L, Lu L, et al. integrated representative single cell RNA sequencing datasets of mouse hearts available from either the GEO databases or Array Express databases. Among the many dynamic changes in multiple cell types, myofibroblasts prominently increased at 7 days post myocardial infarction and a spectrum of signature genes in myofibroblasts were identified. Liu et al. established a list of 260 organelle crosstalk regulators (OCRGs) and analyzed these regulators by searching in GEO databases. Xu et al.. performed massive transcriptomic profiling of endothelial activation by analyzing the GEO databases. Using integrating virtual screening and network pharmacology strategy, Liang et al. proposed Guanxin V as a potential antioxidant in cardiac remodeling with reduced oxidative stress in hydrogen peroxide-stressed cardiomyocytes.

Collectively, this Research Topic assembles a selection of 13 articles, which investigate and overview the novel mechanisms and potential targets for cardiac fibrosis and related diseases associated with different etiologies, introduce a novel animal model for studying HFpEF with cardiac fibrosis, and identify a close relationship between cardiac fibrosis and reduced cardiac performance in aged patients. Further studies applying RNA-Seq, single-cell RNA-Seq, proteomics, metabolomics, and other omics data, together with functional validation experiments and translational researches, will be needed for identifying novel mechanisms and therapeutic targets for cardiac fibrosis and related diseases.

## Author Contributions

YB, HL, and JZ drafted and edited the editorial. All authors contributed to the article and approved the final manuscript.

## Funding

This work is supported by the grants from National Key Research and Development Program of China (2020YFA0803800), Natural Science Foundation of Beijing (7222068), National Natural Science Foundation of China (81970335, 82170285, 92168117, 81770253, and 91849111), Shanghai Rising-Star Program (19QA1403900), Shanghai Committee of Science and Technology (21SQBS00100), and the National Institutes of Heart, USA (HL139748).

## Conflict of Interest

The authors declare that the research was conducted in the absence of any commercial or financial relationships that could be construed as a potential conflict of interest.

## Publisher's Note

All claims expressed in this article are solely those of the authors and do not necessarily represent those of their affiliated organizations, or those of the publisher, the editors and the reviewers. Any product that may be evaluated in this article, or claim that may be made by its manufacturer, is not guaranteed or endorsed by the publisher.
